# Supplementary material for: Carboxylated Carbon Nanotube/Polyimide Films with Low Thermal Expansion Coefficient and Excellent Mechanical Properties
Source: Polymers (Basel). 2022 Oct 27;14(21):4565. doi: 10.3390/polym14214565 (PMC9654399; doi:10.3390/polym14214565)
Supplement: Supplementary file 1 [file polymers-14-04565-s001.zip › polymers-1968067-supplementary.pdf]

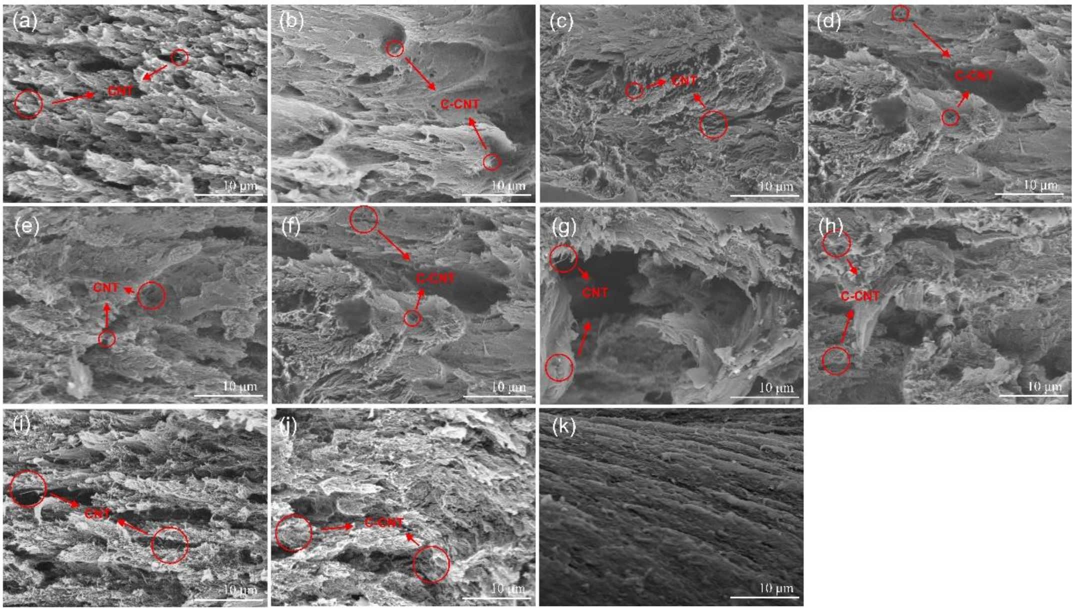

Figure S1. SEM cross-sectional images of (a), (b) 1 wt.% CNT/PI, C-CNT/PI films, (c), (d) 3 wt.% CNT/PI, C-CNT/PI films, (e), (f) 5 wt.% CNT/PI, C-CNT/PI films, (g), (h) 7 wt.% CNT/PI, C-CNT/PI films, (i), (j) 9 wt.% CNT/PI, C-CNT/PI films, (k) pure PI film.
